# Gene-ecology of durum wheat HMW glutenin reflects their diffusion from the center of origin

**DOI:** 10.1038/s41598-018-35251-4

**Published:** 2018-11-16

**Authors:** M. Janni, S. Cadonici, U. Bonas, A. Grasso, A. A. D. Dahab, G. Visioli, D. Pignone, A. Ceriotti, N. Marmiroli

**Affiliations:** 1 Institute of Bioscience and Bioresources (IBBR), National Research Council (CNR), Via Amendola 165/A, 70126 Bari, Italy; 2 Institute of Materials for Electronics and Magnetism (IMEM), National Research Council (CNR), Parco Area delle Scienze 37/A, 43124 Parma, Italy; 3 Department of Chemistry, Life Sciences and Environmental Sustainability, University of Parma, Parco Area delle Scienze, 11/A, 43124 Parma, Italy; 4 National Research Council (CNR), Institute of Agricultural Biology and Biotechnology (IBBA), Via Bassini 15, 20133 Milano, Italy; 5 Regione Emilia-Romagna (IT) SITEIA, PARMA Technopole, Parma, Italy

## Abstract

The production of many food items processed from wheat grain relies on the use of high gluten strength flours. As a result, about 80% of the allelic variability in the genes encoding the glutenin proteins has been lost in the shift from landraces to modern cultivars. Here, the allelic variability in the genes encoding the high molecular weight glutenin subunits (HMW-GSs) has been characterized in 152 durum wheat lines developed from a set of landraces. The allelic composition at the two *Glu-1* loci (*Glu-A1* and -*B1*) was obtained at both the protein and the DNA level. The former locus was represented by three alleles, of which the null allele *Glu-A1c* was the most common. The *Glu-B1* locus was more variable, with fifteen alleles represented, of which *Glu-B1b* (HMW-GSs 7 + 8), -*B1d* (6 + 8) and -*B1e* (20 + 20) were the most frequently occurring. The composition of HMW-GSs has been used to make inferences regarding the diffusion and diversification of durum wheat. The relationships of these allelic frequencies with their geographical distribution within the Mediterranean basin is discussed in terms of gene-ecology.

## Introduction

Wheat is the world’s third most important cereal crop (FAOSTAT 2014, http://faostat3.fao.org) with durum wheat (used primarily for the production of pasta) representing about 5% of global wheat production^1^. The cooking quality of pasta is highly dependent on the flour’s protein content, and in particular on the strength of the gluten^2^, a visco-elastic mass that can be extracted from wheat flour and in which the storage proteins gliadins and glutenins are the main component^3,4^. These proteins comprise approximately 85% of the endosperm’s protein content^5^. The allelic forms of the high molecular weight glutenin subunits (HMW-GSs) are along with LMW major determinant of gluten strength. These subunits are encoded by the *Glu-1* loci, which map to the long arms of the homeologous group 1 chromosomes^6–8^. Each locus comprises a pair of tightly linked genes, one encoding an x- and the other a y-type GS. Thus, the two loci present in durum wheat (*Glu-A1* and *Glu-B1*) in principle encode four GSs but, due to gene silencing, typically only one to three GSs are accumulated in the endosperm^9^. As these proteins have no known biological function other than providing a source of protein to the germinating seedling, their encoding genes show a quite high level of allelic diversity, a finding which has been widely exploited in wheat improvement^10,11^.

According to some estimates, the domestication process reduced the diversity of durum wheat by as much as 84%^12^. Breeding and selection has further narrowed the genetic base of the major crops, so that the genetic diversity represented in gene bank collections is typically much wider than harbored by elite breeding materials^13–16^. Landraces represent a potentially large reservoir of currently unused variation. A collection of some 8,000 durum wheat landraces was assembled by FAO^17^ with a view to avoiding the loss of potentially important diversity and in particular with a view to preserving genes which determine desirable traits no longer retained in modern cultivars^18^. While the relevance to crop improvement of such genetic resources is clear^19^, germplasm collections tend to be greatly underutilized.

A number of studies have addressed the variability in HMW-GSs present in durum wheat^13,20–26^. In some cases, it has been possible to associate a line’s HMW-GS composition with quality parameters and geographical provenance^13–15^. The dispersion of durum wheat is linked to the spread of agriculture through migration from its origin in the Fertile Crescent, westwards into Europe and N. Africa, southwards into Ethiopia and eastwards into central Asia. Specific landraces in durum wheat, as in other crops, have arisen in particular places as a result of both local environmental conditions and local food culture, a manifestation of a co-evolutionary process between humans and plants^27^.

Variation at the HMW-GS loci can be readily visualized electrophoretically separating total proteins extracted from a single grain, either through one dimension (SDS-PAGE) or two (IEF/SDS-PAGE)^7,28^. However, a number of examples have been demonstrated of over-lapping HMW-GSs, leading to an incorrect assignment of alleles^29^. A more precise separation can be achieved by exploiting Lab-on-a-chip technology^30^ or acidic capillary electrophoresis (A-CE)^31^, coupled with a PCR-based analysis to determine variation at the DNA level^32–34^. Here, the HMW-GS composition of a set of 152 lines of durum wheat, developed from a worldwide collection of landraces by imposing single seed descent^16^, has been obtained by conventional SDS-PAGE in conjunction with Lab-on-a-chip technology. Cases where ambiguity remained after this analysis were resolved using a PCR approach^35^.

This paper aims at increase the knowledge on durum wheat domestication and diffusion by analyzing HMW-GS diversity in a set of durum wheat landraces representative of the diversity of this crop. The reason beyond this rests on the linkage between wheat diffusion and human migration from the time the grain become a staple in human feeding.

## Materials and Methods

### Plant material

The germplasm panel comprised a set of 152 lines of durum wheat, developed by single seed descent from a worldwide germplasm collection^16^. The wheat genotypes object of this study, were selected on the basis of a set of image-based morphometric parameters recorded and elaborated for morphological convolution collected in the Italian high throughput phenotyping facility held by ALSIA (Metaponto, Italy). On the basis of these data it was possible to identify a handy set of genotypes (152) representative of the phenotypic variation observed in the original larger collection of 452 genotypes. The parental landraces originated from 31 countries (Table 1). A set of durum and bread wheat cultivars were included to provide standards for the identification of individual GSs^36^ (Suppl. Table 1). Allele designations followed those suggested by Payne *et al*.^37^.

**Table 1:** Provenance of the 152 durum wheat landraces used to develop the germplasm set.

Country of origin	Number of entries
Algeria	6
Balkans*	4
Egypt	4
Ethiopia	10
Former USSR**	4
France	2
Japan	1
Greece	16
India	3
Iran	9
Iraq	13
Italy	13
Libya	2
Mediterranean Islands***	8
Other Middle East****	6
Morocco	9
Perù	1
Iberian Peninsula*****	7
Tunisia	17
Turkey	6
USA	11

*Romania, Bosnia Herzegovina, Bulgaria, Yugoslavia, **Azerbaijan, Russia, Ukraine; ***Cyprus, Crete; ****Syria, Saudi Arabia, Jordan, Afghanistan; *****Spain, Portugal.

### Protein extraction

Total protein was extracted from 30 mg of milled flour of each entry using the sequential procedure described by Singh *et al*.^28^, as modified by Visioli and coworkers^38^. The pellet containing the HMW-GSs was air-dried and the yield determined by dissolving it in a 1:1 mixture of acetonitrile and water containing 0.1% (v/v) trifluoroacetic acid; the quantification of HMW-GSs was obtained using the Bradford method^39^. The samples were finally dried using a Savant SpeedVac SPD1010 device (Thermo Fisher Scientific, Waltham, MA, USA) at 45 °C.

### Protein extraction for use in the Lab-on-a-chip assay

Single grains were ground in a mortar and the resulting samples (~30 mg) extracted in 1 mL dimethyl sulfoxide overnight at room temperature and then twice in 1 mL 50% 2-propanol for 1 h, also at room temperature, to remove the gliadins, albumins and globulins. Between each step, the samples were vortexed for 10 s, then centrifuged (10 min at 14,000 *g*). The resulting pellet was rinsed in 100 μL cold acetone and extracted at 65 °C for 30 min in 200 μL 1% (w/v) SDS solution containing 1% (v/v) dithiothreitol; the samples were finally centrifuged (10 min at 14,000 *g*).

### Allele assignment

The allelic combination at the *Glu-B1* locus was firstly assessed by proteomic analysis (SDS-PAGE and Lab-on-a-chip), then on the basis of the results by a further investigation at a genomic level whenever molecular markers were available, to reach precise and clear assignments for all the SSD genotypes considered in the work. Hence, the final assignment for each allele followed a critical comparison and combination of the results obtained with the three methods.

### SDS-PAGE

Dried samples were suspended in the appropriate volume of loading buffer (Tris HCl 250 mM, pH 6.8; glycerol 50%; SDS 10%; traces of bromophenol blue; β-mercaptoethanol 1:20) to give a concentration of 2.5 µg/µl protein and these were held for 5 min at 95 °C before loading onto 7.5% precast polyacrylamide gels 8,7 × 13,3 cm (L × W) mounted in a Criterion^TM^Dodeca^TM^ Cell device (Biorad, Hercules, CA, USA). Electrophoresis was carried out by passing a constant current of 40 mA for about 2 hours 30 minutes. The gels were fixed in 7% (v/v) glacial acetic acid, 40% (v/v) methanol, then stained overnight in 100% Brilliant Blue (Sigma-Aldrich, Milan, Italy) and de-stained by immersion in deionized water^38^. To increase the resolution of the analyses and to specifically identified *Glu-A1* and *Glu-B1* subunits, the electrophoresis was performed through 12% polyacrylamide gels, following^26^: here the separation was also based on a constant current of 40 mA, but the run time was extended for an additional 3 h once the tracking dye had run off the bottom of the gel. These gels were stained in Coomassie blue R-250 (Biorad).

### Lab-on-a-chip assay

The grain protein extracts were analyzed using a 2100 Bio-analyzer (Agilent Technologies, Palo Alto, CA, USA) equipped with a Protein 230 chip able to resolve proteins in the size range 14–230 kDa. The system was controlled by vB.02.08.SI648 2100 Expert software.

### PCR assays for assessing *Glu-A1* and *Glu-B1* genotype

Genomic DNA was extracted from 100 mg of leaves of each line and of the set of standard cultivars using a GenElute^TM^ Plant Genomic DNA Miniprep kit (Sigma-Aldrich), following the manufacturer’s protocol. A list of the primer pairs used to assay *Glu-A1* and *Glu-B1* and the relevant PCR conditions are given in Suppl. Table 2. The 10 µL PCRs were based on GoTaqHotStart® colorless Mastermix 2X (Promega, Madison, WI, USA) and the reaction conditions replicated those given by^35^. The amplicons were electrophoretically separated through Tris acetate EDTA agarose gels.

### Statistical analysis

Genetic variation at each locus was calculated using the Nei index^13^, H(1 − ∑*p*_*ij*_^2^), where *p*_*ij*_ represented the frequency of the *i*^*th*^ allele at the *j*^*th*^ locus. Allelic frequencies within the panel were determined from that of the alleles in the individual accessions, and then dividing by the 152 genotypes^5^.

### Data accessibility

Essential features are enclosed in the manuscript as Supporting information.

## Results

### *Glu-1* allele diversity

**Figure 1:**
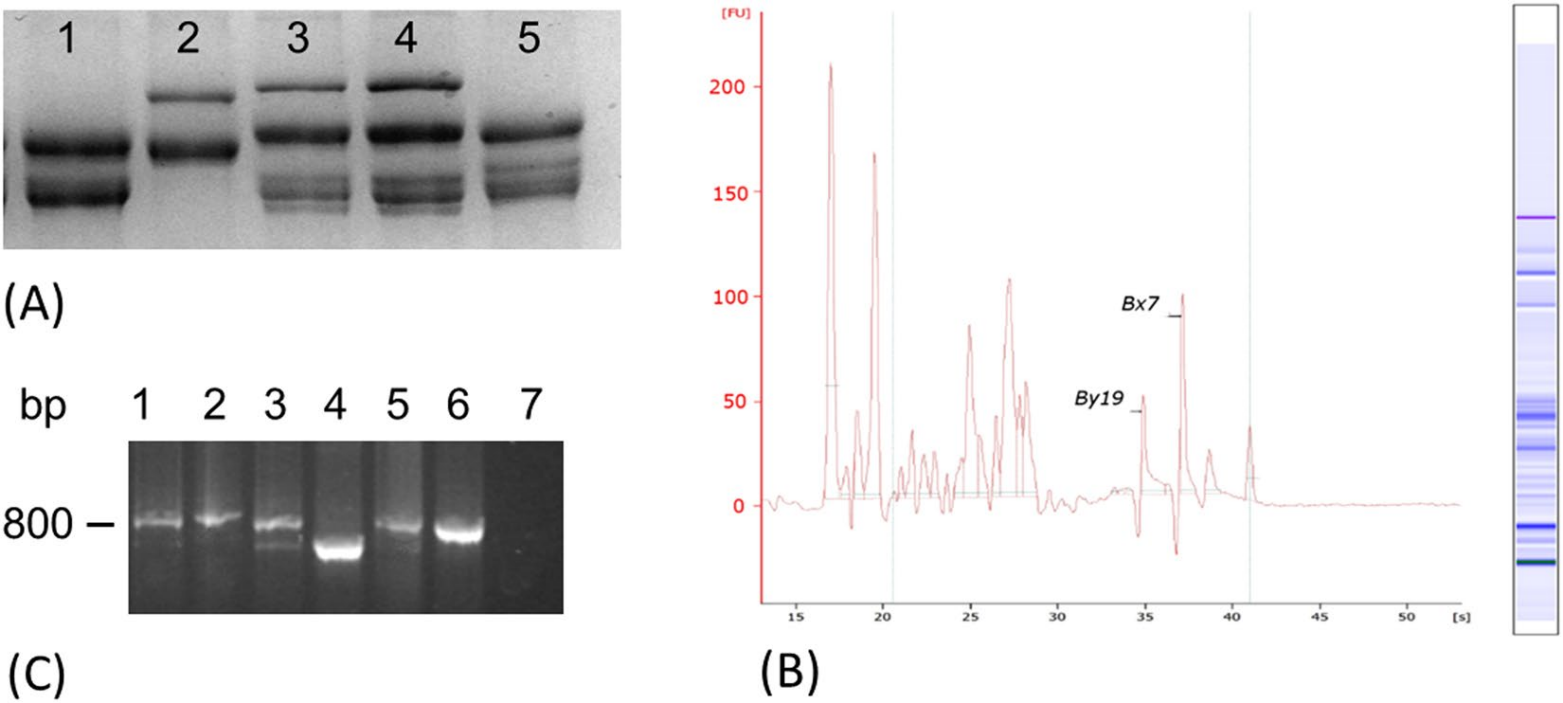
Example of the outputs of the multiple method used to assign the subunit 7 + 19 to the durum wheat landrace SSD 453. SDS-PAGE and Lab-on-a-chip were run on the samples (**A**,**B**). Then PP4 primer pairs (BxF/BxR) were tested (**C**). (**A**) SDS_PAGE; (1) Durazio Rijo cv, 14 + 19; (2) SSD 322, (3) SSD 451, (4) SSD 453, (5) Marques cv, 7 + 15; (**B**) Lab-on-a-chip on SSD 453; (**C**) PP7 marker profile; (1) SSD 451; (2) SSD 453; (3) SSD 494; (4) Francia, 17 + 18, (5) Chinese Spring, 7 + 8; (6) David, 18*; (7) Negative control. To improve the clarity and conciseness of the figure, cropped section of bigger gels have been reported for SDS-PAGE and TAE gel.

The set of HMW-GSs detected in each of the 152 entries is reported in Suppl. Table 2. Three alleles were detected at *Glu-A1* and 15 at *Glu-B1* (Table 3). *Glu-A1* encoded one of two x-type subunits (1, 2*) or carried a null allele, reflecting the presence of, respectively, *Glu-A1a, -A1b* and *-A1c* (Suppl. Table 3). Based on the combined SDS-PAGE and Lab-on-a-chip assays, 107 entries were typed as carriers of *Glu-A1c*, while the other 45 carried either *Glu-A1a* or *-A1b*. The PCR assay was used to reliably distinguish between these latter two alleles: the PP6 and PP7 primer pairs amplify, respectively *-A1b* and *-A1a* (Suppl. Table 2)^35^. The assays suggested that ten of the 45 non-*Glu-A1c* entries carried *Glu-A1b* and the other 35 carried *Glu-A1a* (Table 2). The genotyping exercise slightly increased the frequency of *Glu-A1a* at the expense of *Glu-A1b* over what had been deduced from the SDS-PAGE analysis. The overall diversity at *Glu-A1*, as expressed by H was 0.45. At *Glu-B1*, eleven known alleles (*a*, *b*, *an*, *d*, *e*, *f*, *1 g*, *h*, *z*, *al* and *ak*) were represented, along with four which have not yet been allocated an allelic designation (GS combinations 14 + 19, 14 + 20, 7 + 19 and 6 + 8*, Fig. 1). There were six x-type (7, 6, 20, 13, 14 and 7*) and six y-type (8, 20, 16, 15, 19 and 8*) GSs (Table 2, Suppl. Table 4). The most common GS combinations, such as 7 + 8, 6 + 8 and 20 + 20, were readily identifiable by SDS-PAGE and Lab-on-a-chip method both giving the same result in each case. A further confirmation was assessed applying PP1, PP2, PP3 and PP5 molecular markers. However, when the subunits combinations were not easily distinguishable at the protein level, they were re-analyzed first with Lab-on-a- chip and at the DNA level using the set of PCR assays (Suppl. Table 2, Fig. 1). The allelic constitution of 57 of the 58 entries genotyped in this way was assignable with the exception of SSD 111 (Suppl. Table 3). The application of primer pairs PP1 and PP4 to entries assigned on the basis of SDS-PAGE profiling as carrying *Glu-B1b* (GS 7 + 8) showed that eight rather carried *Glu-B1al* (7 + 8*) and two carried *Glu-B1ak* (7* + 8*). The most frequent alleles were -*B1e* (30.9%), followed by -*B1b* (23.0%) and -*B1d* (18.4%): over 70% of the entries carried one of these three alleles. The most frequently encountered minor alleles were -*B1f* (5.9%), and -*B1al* (5.3%). The non-assigned allele encoding the GS combination 7 + 19 was carried by three entries, while the GS combinations 14 + 20 and 7* + 8* (*Glu-B1ak*) were both present in two entries; finally, the GS combinations 7 + 15 (*Glu-B1z*), 14 + 19, 14 + 15 (*Glu-B1h*) and 6 + 8* were each identified in just a single entry. Five entries produced an x-type but not a y-type *Glu-B1* subunit, of which three carried *Glu-B1a* (GS 7), one produced the *Glu-B1g* (GS 14) and one carried *Glu-B1an* (GS 6) (Table 3). One entry was heterogeneous, carrying both *-B1d* and *-B1e*. The diversity present at *Glu-B1* was considerably greater than at *Glu-A1* (H values of, respectively, 0.80 and 0.45).

**Table 2:** Allele frequency and genetic diversity at the two *Glu-1* loci.

Locus	Allele	Subunit	Number of genotypes	Frequency (%)	H (Nei’s index)
*Glu-A1*	*a*	1	35	23,02	**0**,**45**
	*b*	2*	10	6,57	
	*c*	Null	107	70,39	
*GluB1*	*a*	7	3	1,97	**0**,**803**
	*b*	7 + 8	35	23,02	
	*an*	6	1	0,65	
	*d*	6 + 8	28	18,42	
	*e*	20 + 20	47	30,92	
	*f*	13 + 16	9	5,92	
	*h*	14 + 15	1	0,65	
	—	14 + 19	8	5,26	
	—	14 + 20	2	1,31	
	—	7 + 19	3	1,97	
	*z*	7 + 15	1	0,65	
	*al*	7 + 8*	8	5,26	
	*ak*	7* + 8*	2	1,31	
	—	6 + 8*	1	0,65	
	*lg*	14	1	0,65	
	*abnormal*	7 + 8; 20 + 20	1	0,65	
	undetermined		1	0,65	

— Alleles not annotated.

**Table 3:** The frequency of combined *Glu-A1* and *Glu-B1* genotype within the 152 durum wheat lines.

*Glu-A1* and *Glu-B1* Alleles	*Glu-A1 and* *Glu-B1* Subunits	# of genotypes	Frequencies %
*c, b*	(*null*, 7 + 8)	28	18,42
*c, e*	(*null*, 20 + 20)	26	17,1
*c, d*	(*null*, 6 + 8)	23	15,13
*a, e*	(1, 20 + 20)	20	13,15
*c, f*	(*null*, 13 + 16)	8	5,26
*c, al*	(*null*, 7 + 8*)	7	4,6
*c*, —	(*null*, 14 + 19)	7	4,6
*a, b*	(1, 7 + 8)	5	3,29
*a, d*	(1, 6 + 8)	4	2,63
*c, a*	(*null*, 7)	3	1,97
*b*, —	(2*, 7 + 19)	3	1,97
*b, b*	(2*, 7 + 8)	2	1,31
*a, al*	(1, 7 + 8*)	1	0,66
*b, ak*	(2*, 7* + 8*)	1	0,66
*c, ak*	(*null*, 7* + 8*)	1	0,66
*c, an*	(*null*, 6)	1	0,66
*b, d*	(2*, 6 + 8)	1	0,66
*c*, —	(*null*, 6 + 8*)	1	0,66
*b, e*	(2*, 20 + 20)	1	0,66
*a, f*	(1, 13 + 16)	1	0,66
*c, h*	(*null*, 14 + 15)	1	0,66
*a, 1g*	(1, 14)	1	0,66
*a*, —	(1, 14 + 19)	1	0,66
*c*, —	(*null*, 14 + 20)	1	0,66
*b*, —	(2*, 14 + 20)	1	0,66
*a, z*	(1, 7 + 15)	1	0,66
*a, d* + *e*	(1, 6 + 8/20 + 20)	1	0,66
Undetermined		1	0,66

A summary of the HMW-GS composition of the entries encoded at both *Glu-A1* and *Glu-B1* is presented as Table 3. In all, 27 different combinations were represented across the collection. The four combinations *Glu-A1c/Glu-B1b* (28/152, 18.4%), *Glu-A1c/Glu-B1e* (26/152, 17.1%), *Glu-A1c/Glu-B1d* (23/152, 15.1%) and *Glu-A1a/Glu-B1e* (20/152, 13.2%) predominated. Eight entries carried the combination *Glu-A1c/Glu-B1f*, seven carried *Glu-A1c/Glu-B1al*, seven carried *Glu-A1c/Glu-B1* GS 14 + 19, five carried *Glu-A1a/Glu-B1b*, four carried *Glu-A1a/Glu-B1d*, three carried *Glu-A1c/Glu-B1a*, three carried *Glu-A1b/Glu-B1* GS 7 + 19 and two carried *Glu-A1b/Glu-B1b*. The other 15 GS combinations were each carried by just one entry.

### The geographic distribution of *Glu-1* alleles

**Figure 2:**
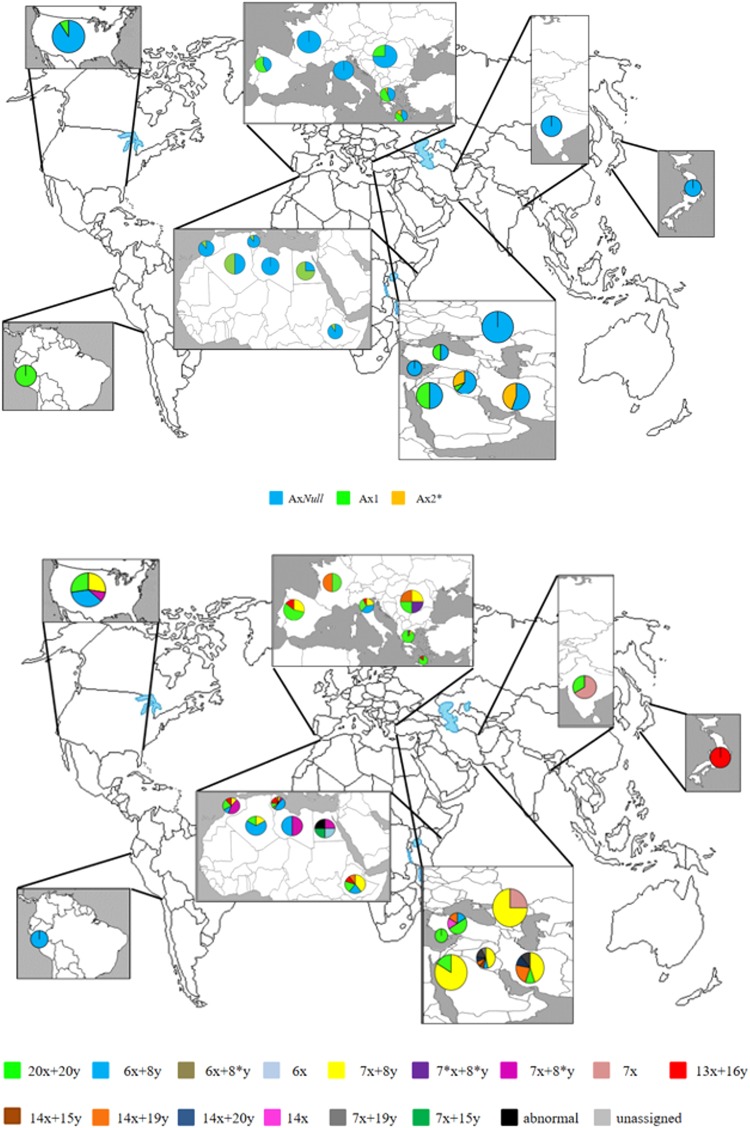
Geographic distribution and the relative frequencies for (**A**) *Glu-A1* and (**B**) *Glu-B1*. Colors indicate the diverse HMW-GS subunits identified within each country of provenance of the durum wheat germplasm entries.

The geographic distribution and the relative frequencies of the various *Glu-A1* and *Glu-B1* alleles across the set of 152 entries are displayed in Fig. 2A,B and Suppl. Table 4. With respect to *Glu-A1*, the most frequently encountered allele in almost all countries of origin (the exceptions were some entries of Balkans, Peru and one from the group of other middle east countries) was *Glu-A1c*, which was present in 107 of the 152 entries. Within the 152 entries, the highest frequencies of the *Glu-A1c* allele were observed among entries originating from Tunisia (9.9%) followed by Italy (8.6%), USA (6.6%), Ethiopia (5.9%), Iraq (5.3%) and Morocco (5.3%). The *Glu-A1a* allele, was detected in 35 of the 152 entries and was particularly frequent in material from S.E. European (Greece: 5.3%, Mediterranean islands: 2.0%, Turkey: 2.0%), Iberian Peninsula (3.0%) or N. African (Egypt and Algeria: 2.0%, Tunisia: 1,3%) provenance. The *Glu-A1b* allele was represented in eight entries originating from S.W. Asia (Iraq and Iran, each 2.6%) and two from Greece (1.3%). With respect to *Glu-B1*, the most frequent allele was *Glu-B1e* (47/152 entries): this allele was concentrated in materials originating from S.E. Europe (Greece, Balkans, Mediterranean Islands), S.W. Asia (Turkey, other middle East countries) and India. S.W. Asia (Iraq, Iran, Syria, and other Middle East countries), India and N. Africa (Morocco, Tunisia, Libya and Egypt) provided the majority of the second most common allele *Glu-B1b* (35/152). These same areas featured a substantial level of diversity at *Glu-B1*, with relative high frequencies of *Glu-B1al* (4.6%), *Glu-B1f* (3.3%) and GS 14 + 19 (1.3%). The *Glu-B1d* allele (28/152) was most strongly associated with a N. African or N. American provenance and was not represented at all among entries originating from around the Black Sea (Turkey) and the Balkans. The *Glu-B1f* allele, although globally rare, was relatively common in entries derived from N. Africa and S. Europe, while the alleles GS 14 + 19, GS 14 + 20 and GS 7 + 19 were encountered in material from S.W. Asia and India (respectively 2.6%, 1.3% and 2.0%). N. American and Italian materials were dominated by carriers of the three high frequency alleles *Glu-B1e, -B1b* and *-B1d*.

## Discussion

Combined assays targeting variation in lines extracted from a set of 152 durum wheat landraces at both the protein and DNA level was able to expose a substantial level of diversity with respect to the HMW glutenins. In addition to the relevance of LMW-GS in determining wheat gluten quality, it has been reported that HMW-GS are also important in increasing gluten polymeric size and thus they contribute to increasing the gluten strength in durum wheat^40^. Their characterization has demonstrated how such a highly heritable trait can serve as a means to trace the diffusion and diversification of a crop species.

Proteomic methods are developing fast, but many still rely on the traditional gel electrophoresis like SDS-PAGE, which includes laborious and time-consuming manual steps and which is difficult to automate. Although resolution can be optimized sizing the gels percentage according to the molecular weight of interest, repeatability in quantitation remains limited. Lab-on-a-chip is a miniaturized electrophoresis based technique for rapid and automated analysis of proteins on a chip, thus shrinking the process which allows to handle small sample volumes increasing the sizing precision and quantitation capability^41^. However, both SDS-PAGE or Lab-on-a-chip, have shown a general overestimation of the apparent molecular sizes of HMW-GS polypeptides when testing cultivars of bread wheat^42^. The combination with DNA molecular markers ensures a more unambiguous assignment for those alleles which were not clearly distinguishable with proteomic methods only.

A comparison of the efficiency and capacity of discrimination of the three methods was described in Goetz *et al*.^41^ and Trad *et al*.^43^ and strengths and limitations of these methods were also reported^29,35,42^.

In this paper, for HMW-GS characterization all three methods were used then the results combined to obtain robust data. SDS-PAGE allowed the alleles to be assigned for 73% of the entries. For 15% (which contained 7* or 8* subunits) DNA molecular markers were required to identify composition, and for the remaining entries (10, 5%) the Lab-on-a-chip in combination with SDS -PAGE techniques were required to correctly identify the alleles. Comparing these techniques, SDS-PAGE was technically the easiest to perform with a reduction of logistic and costs, given previous studies that made use of costly genomic analyses and complex bioinformatics interpretation^44,45^.

The A genome locus *Glu-A1* featured three alleles, of which the null allele was by far the most common, followed by the allele encoding subunit 1; the third allele, responsible for subunit 2*, was represented in only ten lines. The same ranking with respect to allele frequency has been noted by both^46^ in their characterization of 502 durum’s provenance from 23 countries and by Moragues *et al*.^13^ in a study of 63 durum landraces sourced from the Mediterranean Basin. The predominance of the *Glu-A1c* (null) allele has been confirmed in a number of surveys^21,26,47^. The null allele was also the most frequent one when the entries were grouped according to provenance, and was the only allele recovered among the Italian entries. The *Glu-A1a* allele (subunit 1) was relatively frequent in N. African and S.W. Asian material. A frequency imbalance of this degree could be explained where the flour is also destined for bread making, because the presence of the null allele has been correlated with dough extensibility^21^. The presence of the *Glu-A1b* allele (subunit 2*) has been associated with improved performance for some other dough quality parameters (SDS sedimentation value and mixogram score), although this conclusion was reached on the basis of a rather small number of test entries^2,48^.

More extensive variation was present at the *Glu-B1* locus, where 15 alleles were detected; the Moragues *et al*. study identified 14 *Glu-B1* alleles^13^, while the Branlard *et al*. one found ten^46^. The most frequent alleles at this locus were *Glu-B1e, -B1b* and *-B1d*, a ranking consistent with that recorded for a set of 45 Algerian durum wheat landraces and old cultivars^2^. The frequency of each of the minor alleles *Glu-B1f, -B1al* and GS 14 + 19 *(~*5%) was comparable between the present germplasm set and that reviewed by Sissons^2^. The *Glu-B1e* allele has featured strongly in several other germplasm collections^13,15,47^. Both *Glu-B1d* (present in 28 of the 152 lines) and *Glu-B1h* (one line) have been associated with the dough quality parameters SDS sedimentation value and resistance breakdown value^22^, while according to Branlard and coworkers^21^, *Glu-B1d* is also beneficial in terms of biscuit making quality. The high frequency of *Glu-B1b* (23/152 entries) may similarly derive from its association with strong gluten and good pasta quality^15^. According to Sissons^2^, the ranking of *Glu-B1* alleles according to their contribution to pasta quality is -*B1b*>-*B1e*>-*B1d*, an ordering adjusted by Varzakas *et al*.^1^ in order to take into account less common alleles to -*B1i*>*B1g*>-*B1b*>-*B1a*>-*B1d*. The locus was polymorphic in materials originating from the Fertile Crescent, as well as from N. Africa and Ethiopia. The general preponderance of *Glu-B1e* has been noted by other researchers^13,15^, although curiously it is somewhat less ubiquitous in Iberian germplasm^13^. The *Glu-B1f* allele, seen in the African material, was not represented among the Fertile Crescent lines, while some other alleles (GS 7 + 19, -*B1a*, GS 14 + 20, *-B1h*) were present in the latter, but not in the former set of germplasm. The N. African (but not the Ethiopian) lines included representatives of *Glu-B1al*, while the allele encoding -*B1-1g* showed the opposite pattern. GS 14 + 19 was detected in the Fertile Crescent and Ethiopian material, but not in the N. African germplasm. The rare *Glu-B1ak* allele was found only in material with a Balkan or a Greek provenance. A Greek presence in what is now Romania (here represented in the Balkans group) has been dated back as far as the 7^th^ century BCE^49^. At the same time, the evidence is that one of the main routes by which agricultural know-how entered Europe during Neolithic times passed through the Balkans, with Greece representing one of the first European sites where agriculture was adopted^50,51^.

The observed diversity patterns at the two *Glu-1* loci are largely consistent with the idea that durum wheat diversified in three distinct geographical locations, namely the Fertile crescent, N. Africa and the highlands of Ethiopia^44,52,53^. In addition, they support the proposed history of the spread of wheat cultivation across the Mediterranean Basin^15^. The materials originating from the northern and southern shores of the Mediterranean shared a greater degree of genetic similarity than they did with materials of S.W. Asian provenance, which implies that wheat was likely brought to southern Italy from N. Africa^13,54^. The rather rare *Glu-B1a* allele was restricted, as similarly noted by Moragues *et al*.^13^, to India and S.W. Asia, which suggests an independent expansion of wheat cultivation eastwards from the Fertile Crescent. Trading relationships between N. Africa and Europe were undoubtedly encouraged by the geopolitical stability associated with the expansion of the Roman empire. By the beginning of the first millennium, N. Africa had become the source of much of the wheat consumed by Rome^55^. According to Scarascia Mugnozza^56^, one consequence of the occupation of Ethiopia by Italy during the first half of the 20^th^ century was the import of Italian durum wheat germplasm, but the marked differentiation between Italian and Ethiopian landraces exposed by genetic diversity analyses implies that the two gene pools share very little common ancestry^45^.

In summary in this paper we have demonstrated how the application of a proteomic approach and the composition of HMW-GSs may reflects the diffusion and diversification of durum wheat.

## Electronic supplementary material

Supporting information _Janni et al R2

## Data Availability

The data supporting the findings of this study are available from the corresponding authors upon request.
