# Supplementary material for: Gene-ecology of durum wheat HMW glutenin reflects their diffusion from the center of origin
Source: Sci Rep. 2018 Nov 16;8:16929. doi: 10.1038/s41598-018-35251-4 (PMC6240061; doi:10.1038/s41598-018-35251-4)
Supplement: Supplementary file 1 — Supporting information _Janni et al R2 [file 41598_2018_35251_MOESM1_ESM.pdf]

Supplemental Information for:

Gene-ecology of durum wheat HMW glutenin reflects their diffusion from the center of origin

Janni M., Cadonici S., Bonas U., Grasso A., Dahab A. A. D., Visioli G., Pignone D., Ceriotti A., Marmiroli N

Suppl. Table 1. The cultivars used as standards for specific alleles at the two *Glu-1* loci for the purpose of the SDS-PAGE, Lab-on-a-chip and PCR assays. The subunit compositions are based on the Wrigley et al. (2006) classification.

Cultivars name	*Triticum* species	Locus *Glu-1*		
		Glu-A1	*Glu-B1*	*Glu-D1*
Apulia	*Ta*	1	7	2+12
Argelato	*Ta*	*Null*	7	2+12
Biensur	*Td*	*Null*	7+8	/
Chinese Spring	*Ta*	*Null*	7+8	2+12
Simeto	*Td*	*Null*	7+8	/
Svevo	*Td*	*Null*	7+8	/
Abbazia	*Ta*	1	7*+8	2+12
Emilio Morandi	*Ta*	*Null*	7*+8	2+12
Firenze	*Ta*	*Null*	7*+8	2+12
Tiberio	*Ta*	1	7*+8	5+10
Abano	*Ta*	*Null*	7+9	2+12
Adraino	*Ta*	1	7+9	5+10
Pegaso	*Ta*	*Null*	7+9	5+10
Cheyenne	*Td*	2*	7*+9	5+10
Creso	*Td*	*Null*	6+8	/
Dylan	*Td*	*Null*	6+8	/
Florida	*Ta*	1	6+8	5+10
Langdon	*Td*	*Null*	6+8	/
Saragolla	*Td*	*Null*	6+8	/
Est Mottin	*Ta*	1	6*+8*	2+12
Sieve	*Ta*	1	6*+8*	2+12
Tudest	*Ta*	*Null*	6*+8*	2+12*
Virest	*Ta*	1	6*+8*	2+12
Carme	*Ta*	1	18*	2+12
David	*Ta*	1	18*	1+12
Garibaldino	*Ta*	1	18*	2+12
Fabiola	*Ta*	1	17+18	2+12
Francia	*Ta*	2*	17+18	2+12
Duramba	*Td*	2*	13+16	/
Isa	*Td*	*Null*	13+16	/
Cadenza	*Ta*	*Null*	14+15	5+10
Colosseo	*Td*	*Null*	14+15	/
Lobeiro '	*Td*	1	14+15	/

Suppl. Table 1. Continued

Cultivars name	*Triticum* species	Locus *Glu-1*		
		Glu-A1	*Glu-B1*	*Glu-D1*
Capeiti	*Td*	*Null*	20+20	/
Cappelli	*Td*	*Null*	20+20	/
Liberdur	*Td*	*Null*	20+20	/
Lira 42	*Td*	*Null*	20+20	/
Lira 45	*Td*	*Null*	20+20	/
Bagudo	*Td*	1	23+18	/
Durazio Rijo	*Td*	*Null*	14+19	/
Lobeiro	*Td*	1	13+19	/
Marques	*Td*	*Null*	7+15	/
Vermelho Fino	*Td*	1	21	/

Suppl. Table 2. Allelic status at the *Glu-A1* and *Glu-B1* loci of the 152 lines germplasm set.

SSD genotypes	**HMW-GS**		Country of origin
	Glu-A1	*Glu-B1*	
2	*null*	6+8	Algeria
3	*null*	6+8	Algeria
4	*null*	7+8	Iberian Peninsula
6	1	20+20	Algeria
7	*null*	20+20	Iberian Peninsula
11	*null*	7+8*	Tunisia
15	*null*	20+20	Tunisia
17	*null*	13+16	Tunisia
24	*null*	6+8	Tunisia
27	*null*	6+8	Tunisia
28	*null*	6+8	Tunisia
32	*null*	6+8	Tunisia
33	1	20+20	USA-ND
34	*null*	6+8	USA-ND
35	1	6+8	Algeria
36	*null*	6+8	Algeria
41	*null*	6+8	Tunisia
43	*null*	7+8	Tunisia
44	1	6+8	Tunisia
47	*null*	7+8	Former USSR
48	*null*	7+8	Former USSR
49	*null*	7+8	Former USSR
52	*null*	6	Egypt
54	1	**20+20; 7+8**	Egypt
59	1	7+8*	Egypt
63	*null*	7+8	Morocco
64	*null*	7+8*	Morocco
65	*null*	20+20	Morocco
66	*null*	7+8*	Morocco
67	*null*	7+8*	Morocco
68	*null*	7+8*	Morocco
69	*null*	6+8	Morocco
70	*null*	13+16	Morocco
74	*null*	13+16	Japan
83	*null*	6+8	Ethiopia
84	1	7+8	Ethiopia
85	*null*	7+8	Ethiopia
92	*null*	7+8	USA-ND
96	*null*	7	Former USSR
99	*null*	7+8	Ethiopia
102	*null*	14+19	Ethiopia
103	*null*	7+8	USA-MN
105	*null*	14+15	Iraq

Suppl. Table 2. Continued

SSD genotypes	HMW-GS		Country of origin
	Glu-A1	*Glu-B1*	
107	*null*	7+8	Iraq
109	*null*	7+8	Iraq
111	2*	**ND**	Iraq
112	*null*	7+8	Iraq
113	*null*	7+8	Iraq
114	*null*	7+8	Iraq
115	*null*	7+8	Iraq
116	2*	6+8	Iraq
118	*null*	7+8	Balkans
120	*null*	7+8	USA-WA
122	*null*	6+8	USA-ND
123	*null*	20+20	USA-MN
124	*null*	6+8	USA-ND
125	*null*	20+20	USA-ND
128	*null*	7+8*	USA-ND
131	*null*	7+8	Italy
135	*null*	20+20	Turkey
136	1	20+20	Turkey
137	*null*	14+19	Turkey
142	1	20+20	Iberian Peninsula
146	1	20+20	Iberian Peninsula
147	1	13+16	Iberian Peninsula
155	*null*	7	India
158	*null*	7+8	Ethiopia
168	*null*	6+8	Turkey
171	1	6+8	Perù
173	1	7+15	Egypt
178	*null*	14+19	France
180	*null*	7	India
182	*null*	20+20	India
185	1	20+20	Turkey
188	1	7+8	Other Middle East
191	*null*	7+8	Other Middle East
195	1	7+8	Other Middle East
240	*null*	20+20	Ethiopia
243	*null*	13+16	Ethiopia
244	*null*	20+20	Ethiopia
246	*null*	6+8	Ethiopia
253	*null*	20+20	Mediterranean Islands
255	1	7+8	Other Middle East
256	2*	14+20	Iran
262	*null*	20+20	Iran
264	*null*	6+8	Italy

Suppl. Table 2. Continued

SSD genotypes	HMW-GS		Country of origin
	Glu-A1	*Glu-B1*	
266	*null*	14+19	Iran
269	2*	7+8	Iran
271	2*	7+8	Iran
278	1	20+20	Balkans
283	*null*	20+20	France
288	1	7+8	Algeria
290	*null*	6+8*	Tunisia
292	*null*	13+16	Tunisia
294	*null*	13+16	Tunisia
298	1	6+8	Tunisia
302	*null*	14+19	Tunisia
303	*null*	20+20	Tunisia
308	*null*	6+8	Tunisia
315	*null*	7+8*	Libya
322	1	14	Turkey
325	*null*	7+8	Other Middle East
326	*null*	14+19	Balkans
328	*null*	7+8	Iberian Peninsula
330	1	20+20	Morocco
335	*null*	14+20	Iraq
336	1	14+19	Iraq
338	*null*	7+8	Iran
343	2*	7+19	Iran
345	*null*	20+20	Other Middle East
348	*null*	14+19	Iran
350	*null*	7+8	Iran
393	*null*	7*+8*	Balkans
397	1	20+20	Mediterranean Islands
399	1	20+20	Mediterranean Islands
400	1	20+20	Mediterranean Islands
407	*null*	20+20	Greece
409	1	20+20	Greece
411	1	20+20	Greece
412	*null*	20+20	Greece
414	*null*	20+20	Greece
415	*null*	20+20	Mediterranean Islands
416	*null*	20+20	Greece
421	*null*	13+16	Mediterranean Islands
422	*null*	20+20	Mediterranean Islands
423	1	20+20	Greece
424	1	20+20	Greece
426	1	20+20	Greece
427	1	20+20	Greece

Suppl. Table 2. Continued

SSD genotypes	HMW-GS		Country of origin
	Glu-A1	*Glu-B1*	
431	1	20+20	Greece
432	1	20+20	Greece
441	2*	20+20	Mediterranean Islands
443	*null*	7+8	Greece
447	*null*	20+20	Italy
451	2*	7+19	Iraq
453	2*	7+19	Iraq
457	*null*	7+8	Italy
459	*null*	6+8	USA
467	*null*	20+20	Greece
470	1	20+20	Iberian Peninsula
487	*null*	20+20	Greece
494	2*	7*+8*	Greece
499	*null*	20+20	Italy
500	*null*	20+20	Italy
511	*null*	6+8	Libya
513	*null*	20+20	Italy
526	*null*	13+16	Italy
531	*null*	6+8	Italy
532	*null*	6+8	Italy
533	*null*	6+8	Italy
533bis	*null*	6+8	Italy
534	*null*	7+8	Italy

ND Not Determined

Suppl. Table 3. PCR primer combinations and reaction conditions used to generate *Glu-1* genotypes.

**Primer pairs n°**	**Primers pair name**	***Glu*-1 alleles identified**	**Fragment size (bp)**	**Tm**	**References**
PP1	ZSBy8_F5/ ZSBy8_R5	By8/By8*	527 /no band	64 °C	[30,33]
PP2	ZSBy9_F2/ ZSBy9_R2	By16	280+350+ 400	62 °C	[30]
		By20	no bands		
		By8,By8*,By9,By15,By18	280+ 350		
		By18/By26	280		
PP3	ZSBy9_aF1/ZSBy9_aR3	By9/By20	662/720	59 °C	[30,33]
PP4	Bx_F/ Bx_R	Bx17/No Bx17	669/670+770	58 °C	[31,33]
		Bx7*	650+750		
		Bx7	800		
PP5	Bx7_F/Bx7_R	Bx6	250	50 °C	[56]
		Bx7,Bx17	220		
PP6	Ax_F/Ax2*_R	Ax1/Ax2*	no bands/1319	58 °C	[31]
PP7	Ax1/Ax2*_C<br>Ax1/Ax2*_D	Ax1/Ax2*	1500/1400	60 °C	[57]

PP: Primer Pair; Tm: Melting Temperature

Suppl. Table 4. Allele frequency at *Glu-A1* and *Glu-B1* for entries grouped by provenance.

Locus	Allele	Total[$] (152)	Other Middle East[#] (Tot. 6)	Total[$] (152)	Algeria[#] (Tot. 6)	Total[$] (152)	Ethiopia[#] (Tot. 10)	Total[$] (152)	France[#] (Tot. 2)	Total[$] (152)	Japan[#] (Tot. 1)	Total[$] (152)	Egypt[#] (Tot. 4)
Glu-A1						0,65	10	/	/	/	/		
	1	1,97	50	1,97	50	/	/	/	/	/	/	1,97	75
	2*	/	/	/	/	5,92	90	1,31	100	0,65	100	/	/
	null	1,97	50	1,97	50							0,65	25
Glu-B1						/	/	/	/	/	/		
	7	/	/	/	/	2,63	40	/	/	/	/	/	/
	7+8	3,28	83,3	0,65	16,67	/	/	/	/	/	/	/	/
	6	/	/	/	/	1,31	20	/	/	/	/	0,65	25
	6+8	/	/	2,63	66,66	1,31	20	0,65	50	/	/	/	/
	20+20	0,65	16,4	0,65	16,67	0,65	10	/	/	0,65	100	/	/
	13+16	/	/	/	/	/	/	/	/	/	/	/	/
	14+15	/	/	/	/	0,65	10	0,65	50	/	/	/	/
	14+19	/	/	/	/	/	/	/	/	/	/	/	/
	14+20	/	/	/	/	/	/	/	/	/	/	/	/
	7+8*	/	/	/	/	/	/	/	/	/	/	0,65	25
	7*+8*	/	/	/	/	/	/	/	/	/	/	/	/
	6+8*	/	/	/	/	/	/	/	/	/	/	/	/
	7+19	/	/	/	/	/	/	/	/	/	/	/	/
	7+15	/	/	/	/	/	/	/	/	/	/	0,65	25
	14	/	/	/	/	/	/	/	/	/	/	/	/
	abnormal	/	/	/	/	/	/	/	/	/	/	0,65	25
	unknown	/	/	/	/							/	/

[$]: Frequencies of the alleles considering the total 152 SSD genotypes analyzed.
[#]: Percentage of the single allele within the entries of the country.

Suppl. Table 4. Continued

Locus	Allele	Total$ (152)	Greece# (Tot. 16)	Total$ (152)	India# (Tot. 3)	Total$ (152)	Iran# (Tot. 9)	Total$ (152)	Iraq# (Tot. 13)	Total$ (152)	Italy# (Tot. 13)	Total$ (152)	Mediterranean Islands# (Tot. 8)
Glu-A1													
	1	5,26	50	/	/	/	/	0,65	7,7	/	/	1,97	37,5
	2*	0,65	6,25	/	/	2,63	44,44	2,63	30,76	/	/	0,65	12,5
	null	4,6	43,75	1,97	100	3,29	55,56	5,26	61,54	8,55	100	2,63	50
Glu-B1													
	7	/	/	1,31	66,67	/	/	/	/	/	/	/	/
	7+8	0,65	6,25	/	/	2,63	44,44	3,95	46,15	1,97	23,08	/	/
	6	/	/	/	/	/	/	/	/	/	/	/	/
	6+8	/	/	/	/	/	/	0,65	7,7	3,29	38,46	/	/
	20+20	9,21	87,5	0,65	33,33	0,65	11,11	/	/	2,63	30,77	4,6	87,5
	13+16	/	/	/	/	/	/	/	/	0,65	7,69	0,65	12,5
	14+15	/	/	/	/	/	/	0,65	7,7	/	/	/	/
	14+19	/	/	/	/	1,31	22,22	0,65	7,7	/	/	/	/
	14+20	/	/	/	/	0,65	11,11	0,65	7,7	/	/	/	/
	7+8*	/	/	/	/	/	/	/	/	/	/	/	/
	7*+8*	0,65	6,25	/	/	/	/	/	/	/	/	/	/
	6+8*	/	/	/	/	/	/	/	/	/	/	/	/
	7+19	/	/	/	/	0,65	11,11	1,31	15,38	/	/	/	/
	7+15	/	/	/	/	/	/	/	/	/	/	/	/
	14	/	/	/	/	/	/	/	/	/	/	/	/
	abnormal	/	/	/	/	/	/	/	/	/	/	/	/
	unknown	/	/	/	/	/	/	0,65	7,7	/	/	/	/

$: Frequencies of the alleles considering the total 152 SSD genotypes analyzed.
#: Percentage of the single allele within the entries of the country.

Suppl. Table 4. Continued

Locus	Allele	Total$ (152)	Libya# (Tot. 2)	Total$ (152)	Morocco# (Tot. 9)	Total$ (152)	Peru# (Tot. 1)	Total$ (152)	Former USSR# (Tot.4)	Total$ (152)	Balkans# (Tot. 4)
Glu-A1											
	1	/	/	0,65	11,11	0,65	100	/	/	0,65	25
	2*	/	/	/	/	/	/	/	/	/	/
	null	1,31	100	5,26	88,89	/	/	2,63	100	1,97	75
Glu-B1											
	7	/	/	/	/	/	/	0,65	25	/	/
	7+8	/	/	0,65	11,11	/	/	1,97	75	0,65	25
	6	/	/	/	/	/	/	/	/	/	/
	6+8	0,65	50	0,65	11,11	0,65	100	/	/	/	/
	20+20	/	/	1,31	22,22	/	/	/	/	0,65	25
	13+16	/	/	0,65	11,11	/	/	/	/	/	/
	14+15	/	/	/	/	/	/	/	/	/	/
	14+19	/	/	/	/	/	/	/	/	0,65	25
	14+20	/	/	/	/	/	/	/	/	/	/
	7+8*	0,65	50	2,63	44,45	/	/	/	/	/	/
	7*+8*	/	/	/	/	/	/	/	/	0,65	25
	6+8*	/	/	/	/	/	/	/	/	/	/
	7+19	/	/	/	/	/	/	/	/	/	/
	7+15	/	/	/	/	/	/	/	/	/	/
	14	/	/	/	/	/	/	/	/	/	/
	abnormal	/	/	/	/	/	/	/	/	/	/
	unknown	/	/	/	/	/	/	/	/	/	/

$: Frequencies of the alleles considering the total 152 SSD genotypes analyzed.
#: Percentage of the single allele within the entries of the country.

Suppl. Table 4. Continued

Locus	Allele	Total[$] (152)	Iberian Peninsula[#] (Tot. 7)	Total[$] (152)	Tunisia[#] (Tot. 17)	Total[$] (152)	Turkey[#] (Tot. 6)	Total[$] (152)	USA[#] (Tot. 11)
Glu-A1									
	1	2,63	57,2	1,31	11,76	1,97	50	0,65	9,1
	2*	/	/	/	/	/	/	/	/
	null	1,97	42,8	9,87	88,24	1,97	50	6,58	90,9
Glu-B1									
	7	/	/	/	/	/	/	/	/
	7+8	1,31	28,6	0,65	5,89	/	/	1,97	27,3
	6	/	/	/	/	/	/	/	/
	6+8	/	/	5,26	47,05	0,65	16,67	2,63	36,3
	20+20	2,63	57,1	1,31	11,75	1,97	50	1,97	27,3
	13+16	0,65	14,3	1,97	17,64	/	/	/	/
	14+15	/	/	/	/	/	/	/	/
	14+19	/	/	0,65	5,89	0,65	16,67	/	/
	14+20	/	/	/	/	/	/	/	/
	7+8*	/	/	0,65	5,89	/	/	0,65	9,1
	7*+8*	/	/	/	/	/	/	/	/
	6+8*	/	/	0,65	5,89	/	/	/	/
	7+19	/	/	/	/	/	/	/	/
	7+15	/	/	/	/	/	/	/	/
	14	/	/	/	/	0,65	16,67	/	/
	abnormal	/	/	/	/	/	/	/	/
	unknown	/	/	/	/	/	/	/	/

[$]: Frequencies of the alleles considering the total 152 SSD genotypes analyzed.
[#]: Percentage of the single allele within the entries of the country.
